# SEOM clinical guidelines for pancreatic and biliary tract cancer (2020)

**DOI:** 10.1007/s12094-021-02573-1

**Published:** 2021-03-03

**Authors:** Mª A. Gómez-España, A. F. Montes, R. Garcia-Carbonero, T. M. Mercadé, J. Maurel, A. M. Martín, R. Pazo-Cid, R. Vera, A. Carrato, J. Feliu

**Affiliations:** 1 Medical Oncology Department, Hospital Universitario Reina Sofía, IMIBIC, CIBERONC, Córdoba, Spain; 2 Medical Oncology Department, Complexo Hospitalario Universitario de Ourense (CHUO), Orense, Spain; 3 Medical Oncology Department, Hospital Universitario, UCM, CNIO, CIBERONC, 12 de Octubre, IIS imas12, Madrid, Spain; 4 Medical Oncology Department, Hospital Universitari Vall D´Hebron, Barcelona, Spain; 5 Medical Oncology Department, Hospital Clinic Barcelona, Barcelona, Spain; 6 Medical Oncology Department, Hospital General Universitario Gregorio Marañón, Madrid, Spain; 7 Medical Oncology Department, Hospital Universitario Miguel Servet, Zaragoza, Spain; 8 Medical Oncology Department, Complejo Hospitalario de Navarra, Pamplona, Spain; 9 Medical Oncology Department, Hospital Universitario Ramón Y Cajal, Alcala University, IRYCIS, CIBERONC, Madrid, Spain; 10 Medical Oncology Department, Hospital Universitario La Paz, CIBERONC, IDIPAZ, Madrid, Spain

**Keywords:** Pancreatic cancer, Biliary tract cancer, Treatment, Chemotherapy, Radiotherapy

## Abstract

Pancreatic cancer (PC) and biliary tract cancer (BTC) are both aggressive and highly fatal malignancies. Nowadays we have a profound knowledge about the molecular landscape of these neoplasms and this has allowed new therapeutic options. Surgery is the only potentially curative therapy in both cancers, but disease recurrence is frequent. In PC, adjuvant treatment with mFOLFIRINOX has improved overall survival (OS) and in BTC adjuvant treatment with capecitabine seems to improve OS and relapse-free survival. Concomitant radio-chemotherapy could also be considered following R1 surgery in both neoplasms. Neoadjuvant treatment represents the best option for achieving an R0 resection in borderline PC. Upfront systemic chemotherapy is the treatment of choice in unresectable locally advanced PC and BTC; then locoregional therapy could be considered after an initial period of at least 3–4 months of systemic chemotherapy. In metastatic PC, FOLFIRINOX or Gemcitabine plus nab-paclitaxel have improved OS compared with gemcitabine alone. In metastatic BTC, cisplatin plus gemcitabine constitute the standard treatment. Progress in the knowledge of molecular biology has enabled the identification of new targets for therapy with encouraging results that could in the future improve the survival and quality of life of patients with PC and BTC.

## Methodology

The present guidelines have been based on the relevant studies published and with the consensus of the all authors. In order to assess the level and quality of evidence and to establish a grade of recommendation of the different statements in this guideline, we based ourselves on The Infectious Diseases Society of America-US Public Health Service Grading System.

## Pancreatic cancer

### Introduction

Pancreatic ductal adenocarcinoma (PDAC) is the third cause of death by cancer at the European Union. It will become the second during the next 10 years. Only 5% of PDAC patients survive 10 years. Its incidence is raising and is usually diagnosed at an advanced stage due to its particular aggressive biology and non-specific symptoms [1]. Only 15–20% of patients are candidates for surgery at presentation and two thirds of them recur. Treatment usually consists of chemotherapy combinations with no targeted agents available. There is a desperate need for an earlier diagnosis to identify the population at a higher PDAC risk for developing primary and secondary prevention programs, a better policy making and for effective medical treatments. Patients diagnosed with PDAC should undergo an assessment of risk for hereditary syndromes known to be associated with an increased risk for pancreatic adenocarcinoma.

### Epidemiology and risk factors

Age, tobacco smoking, alcohol consumption, overweight, new onset diabetes, pancreatitis, etc. are associated with a slight increase in the incidence of PDAC. Familial cancer with pathogenic germline alterations (*BRCA1, BRCA2, ATM, PALB2, MLH1, MSH2, MSH6, PMS2, CDKN2A, P53* and others still unknown), which involves 10% of the whole PDAC patients, is the only established high-risk population. Integration of omics data with clinical factors could design a signature for PDAC risk scores. A blood-based early diagnosis in the high-risk subgroup is the most promising approach.

### Diagnosis

A PDAC-oriented CT scan may show a pancreatic mass in most of cases. When the tumor is iso-dense with the stroma, an MRI is needed. More than 50% of PDAC pathology diagnosis is performed from a fine needle aspiration (FNA) cytology of the primary tumor, with no architectural tissue features, in cancer with a dense stroma. Diagnosis accurateness could be improved by a Tru-cut needle biopsy obtained from a liver metastasis, at the surgery of the primary tumor or through a thick-enough trans-duodenal biopsy.

#### Molecular biology

Pre-neoplastic lesions (cystic tumors, intraductal papillary-mucinous neoplasms -IPMN, and pancreatic intraepithelial neoplasia-PanIN) show different carcinogenic pathways conditioned by the cell of origin and the genetic and microenvironment alterations observed. They should be classified according to imaging features and novel biomarkers.

The original cell giving rise to PDAC is controversial as some acinar cells can experience acinar to ductal metaplasia (ADM) under stress conditions. *KRAS* mutations make the pancreatic ductal cells as well as these ADM cells to remain ductal, upregulate *EGFR* signaling, and progress to PanIN1. The evolution to PanIN2 needs additional mutations in tumor suppressor genes like *CDKN2A*. Inactivation of other suppressor genes like SMAD4, BRCA2, TP53, etc. occurs at PanIN3 or high-grade dysplasia, considered as carcinoma in situ, and in PDAC. After *BRG1* inactivation, *KRAS* mutated duct cells may also progress to IPMN and some of them to invasive IPMN or PDAC through consecutive gene alterations.

Cellular heterogeneity is shown in preneoplastic lesions. Cellular plasticity at early stages is corroborated by their presence in the circulation and at secondary organs before progression to carcinoma [2].

In parallel, the microenvironment converts to proinflammatory, there is a crucial crosstalk with cancer cells favoring the development of preneoplastic lesions and finally PDAC in a desmoplastic, fibrotic, and immune-suppressive stroma with a loose vasculature and poor tumor perfusion leading to hypoxia and suboptimal drug and immune cells entrance.

The most common driver mutated genes in PDAC are *KRAS*, *TP53*, *CDKN2A* and *SMAD4*. Unfortunately, these pathways are still not therapeutically targeted. Epigenetic changes and metabolic rewiring are related to metastatic behavior*.*

Tumor-associated macrophages (TAM-M2), cancer-associated fibroblasts (CAF) Tregs and myeloid-derived suppressor cells (MDSCs) inhibit the immune.

Transcriptomics classify PDAC in basal-cell tumors, also called squamous, which survive shorter than the classic subtypes (immunogenic, pancreatic progenitor and ADEX) [3]. Low GATA6 expression [4] and high KRT14 or KRT5/6 [5] are surrogate biomarkers of basal-cell tumors that are less differentiated and associated with an activated stroma. They may respond better to gemcitabine-nab-paclitaxel than to FOLFIRINOX while classical type tumors respond better to FOLFIRINOX. Serine hydrolase carboxylesterase 2 (CES2) is predictive of response to irinotecan as mediates its intra-tumoral activation. It is associated with type 2 diabetes [6].

The digestive microbiome has demonstrated multiple effects on tumor biology, and bacteria, mostly located intracellularly in both cancer and immune cells, may be related to the different phenotypes and on their interaction with the immune system [7].

### Staging

After a suspicion of pancreatic cancer (weight loss, jaundice, pain) diagnosis should include a complete anamnesis with performance status evaluation, laboratory test (including CA19.9, reactive C protein and albumin levels) and in patients (≥ 70 years) frailty assessment.

Pathologic diagnosis is recommended in resectable disease and mandatory in borderline-resectable, locally advanced, and metastatic disease. Endoscopic ultrasonography (EUS) and fine-needle aspiration (FNA) is the optimal technique to obtain pathologic diagnosis in localized disease and should be done before biliary stent placing. Thoracic and abdominal three-phase (pancreatic, arterial and portal) multidetector row computed tomography (MDCT) with 2–3 mm/thickness should be done in all cases for staging. Radiological report should include tumour vascular [venous (superior mesenteric vein and portal vein) and arterial (celiac trunk, superior mesenteric artery and common hepatic artery)] involvement (see Table 1). Liver MRI, PET-CT and laparoscopy, can complement staging.

**Table 1:** Criteria defining resectability status in JPS classification 7th edition, and NCCN 2016

	JPS classification 7th edition (2016)	NCCN 2016
Resectable: R	SMV/PV: no tumor contact or contact of less than 180° without occlusion SMA, CA, CHA: no tumor contact/invasion	SMV/PV: no tumor contact, or contact of less than 180° without vein contour irregularity SMA, CA, CHA: no arterial tumor contact
Borderline resectable: BR	Subclassified according to SMV/PV invasion alone or arterial invasion	No subclassification according to SMV/PV invasion alone or arterial invasion
BR-PV (SMV/PV invasion alone)	SMV/PV: tumor contact/invasion of 180° or more/occlusion, not exceeding the inferior border of the duodenum SMA, CA, CHA: no tumor contact/invasion	SMV/PV: solid tumor contact of 180° or more, contact of less than 180° with contour irregularity of the vein or thrombosis of the vein but with suitable vessel proximal and distal to the site of involvement allowing for safe and complete resection and vein reconstruction IVC: solid tumor contact
BR-A (arterial invasion)	SMA, CA: tumor contact/invasion of less than 180° without showing stenosis/deformity CHA: tumor contact/invasion without showing tumor contact/invasion of the PHA and/or CA. (In case of contact/invasion to both portal vein and peripancreatic arteries, it was graded as BR-A.)	Pancreatic head/uncinate process:SMA: solid tumor contact of less than 180°. CHA: solid tumor contact without extension to CA/hepatic artery bifurcation allowing for safe and complete resection and reconstruction. Presence of variant arterial anatomy (RHA, CHA) and the presence of tumor contact as it may affect surgical planning Pancreatic body/tail:CA: solid tumor contact of less than 180°CA: solid tumor contact of 180 or more degree without the involvement of the aorta and with intact and uninvolved GDA (some members prefer this criteria to be in the UR category)
UR-LA (locally advanced)	SMV/PV: tumor contact/invasion of 180 or more degree/occlusion, exceeding the inferior border of the duodenum SMA, CA: tumor contact/invasion of 180 or more degree CHA: tumor contact/invasion showing tumor contact/invasion of the PHA and/or CA AO: tumor contact or invasion	Venous. Head/uncinate process: SMV/PV: unreconstructable due to tumor involvement/occlusion Contact with most proximal draining jejunal branch into SMV Body and tail SMV/PV: unreconstructable due to tumor involvement/occlusion Arterial. Head/uncinate process: SMA, CA: solid tumor contact of 180 or more degree. Solid tumor contact with the 1st jejunal SMA branch Body and tail. SMA, CA: solid tumor contact of 180 or more degree. Solid tumor contact with the CA and aortic involvement
UR-M	Distant metastasis including non-regional lymph node metastasis	Distant metastasis (including non-regional lymph node metastasis)

*SMV* superior mesenteric vein, *PV* portal vein, *SMA* superior mesenteric artery, *CA* celiac artery, *CHA* common hepatic artery, *PHA* proper hepatic artery *RHA* right hepatic artery

**Recommendations**

Endoscopic ultrasonography (EUS) and fine needle aspiration (FNA) and thoracic and abdominal three-phase (pancreatic, arterial and portal) MDCT are the standard procedures. EUS-FNA should be done before biliary stent placing (III, A).

### Treatment

#### Resectable disease

Twenty percent of patients have resectable disease based on MDCT and up-front surgical resection is indicated. Treatment decision should be done only after careful evaluation in a multidisciplinary dedicated team. Surgical resection should be done preferably in high-volume Centers (recommended > 20 pancreatic procedures/year) [8]. Pancreatic resections in specialized institutions have mortality rates below 5%.

Despite adequate oncologic resections, 76–82% of patients have positive margins (R1) if pathological specimens are evaluated with standardized pathological procedures based on the Leeds Pathology protocol [9]. Recurrence rate at 2 year occurs in 70–80% of patients (either local and distant recurrences).

**Recommendations**

Surgical resection should be done preferably in high-volume specialized Centers. Pathological specimen should be evaluated with standardized pathological procedures based on the Leeds Pathology protocol (III, A).

CONKO-1 demonstrated superiority of gemcitabine over no therapy and fulfil primary end-point (increase 6 months median disease-free survival) and ESMO-MCBS [10] (7.5% 3 year DFS in the control group and 23.5% in gemcitabine group).

The ESPAC-4 trial compared adjuvant gemcitabine vs. gemcitabine combined with capecitabine [11]. 3 year relapse-free survival was 20.9% in the gemcitabine group and 23.8% in the gemcitabine-capecitabine group with a HR 0.86 that did not fulfill pre-defined primary end-point and ESMO-MCBS [10].

Recently the PRODIGE group compared adjuvant gemcitabine vs. modified FOLFIRINOX (mFOLFIRINOX) in resected patients with ECOG PS 0–1 and CA19.9 below 180 U/ml. The study fulfils pre-defined primary end-point (> 10% difference in PFS at 3 years) and ESMO-MCBS strict criteria (3 year PFS of 39.7% vs. 21.6% and a HR. 0.58). Although patients under 79 years were included, only 20% of all patients have > 70 years. Frailty assessment is recommended, before treat patients over 70 years with mFOLFIRINOX [12].

Finally, PREOPANC study compared preoperative chemo-radiotherapy with gemcitabine vs. standard adjuvant gemcitabine alone. This study included both, resectable and border-line resectable patients. The primary end-point was designed to improve median survival (from 11 to 17 months). Unfortunately, PREOPANC did not fulfill primary end-point and ESMO-MBSC criteria [13].

**Recommendations**

Adjuvant gemcitabine is recommended, in medium-fit ECOG PS 0–1 patients older than 70 years. In patients < 70 years and ECOG PS 0–1 and fit patients between 70 and 80 years adjuvant therapy with mFOLFIRINOX could be considered standard treatment (I, A).

#### Borderline disease

Numerous non-randomized studies in patients with borderline disease (BR) that have used different treatment regimens with neoadjuvant chemotherapy or chemoradiation have managed to increase the resectability and R0 resection rate rates in BR. A korean randomized phase 2/3 trial test the neoadjuvant strategy in BR patients. The trial included 110 patients that were randomized to be treated with gemcitabine-based neoadjuvant chemoradiation treatment versus surgery upfront. The trial demonstrated superior 2 years OS in the neoadjuvant arm (40.7% vs. 26.1%, HR 1.45, *p *= 0.0028). R0 resection rate was also significantly higher in the neoadjuvant group (51.8% vs. 26.1%, *p* = 0.004) [14]. There is limited evidence to recommend specific neoadjuvant regimens off-study, and practices vary with regard to the use of chemotherapy and chemoradiation [15].

SWOG1505, a randomized phase II trial, demonstrated no difference between the perioperative treatment with mFOLFIRINOX or gemcitabine with albumin-bound paclitaxel [16].

**Recommendations**

1. Patients with BR should be included in Clinical Trials wherever possible.
2. FOLFIRINOX/mFOLFIRINOX and gemcitabine in combination with albumin-bound paclitaxel are two acceptable regimens to be used in the neoadjuvant strategy (II, B).
3. Chemoradiotherapy with gemcitabine or capecitabine is an option (I, C).

#### Locally advanced disease

Owing to the difficulty of relying on strict criteria for unresectability, it should be recommended that all locally advanced pancreatic cancer (LAPC) are discussed by a multidisciplinary board, and that these evaluations are repeated after treatment induction to confirm definitive unresectability. The possible role for surgery has been extended recently to some selected LAPC. A new classification may help the multidisciplinary boards to define the optimal strategy and the goal of the treatment. Type A are tumors that may be considered for surgery after induction chemotherapy, and type B are definitively unresectable tumors [17]. The objective in unresectable LAPC is to increase OS and quality of life, maintaining local control of the disease.

The initial approach is controversial. The LAP07 study showed no benefit in survival when comparing RT-CT vs. chemotherapy (CT) in patients with LAPC and controlled after 4 months of induction QT treatment disease, but showed better local control and increased free survival progression [18]. The standard CT treatment in LAPC is gemcitabine, but the significant increase in efficacy with new schedules in metastatic disease (FOLFIRINOX for PS 0–1 [19] and gemcitabine/albumin-bound paclitaxel for PS 0–2 [20]) has lead to their use, also in LAPC, as a reasonable alternative. However, we only have data from observational, pooled analysis or small phase II trials. The duration of the initial treatment is not established and depends on tolerability and tumor response. Using chemoradiotherapy consolidation in patients with response or stabilization after 4–6 months of chemotherapy is an option to consider, vs. maintenance treatment with chemotherapy in patients with good PS. In this context, capecitabine showed a better toxicity profile and efficacy than gemcitabine [21].

**Recommendations**

1. LAPC has to be discussed in a multidisciplinary tumor board in order to define its resectability (IV, A).
2. The standard treatment for LAPC is chemotherapy (I, A).
3. By extrapolation in the metastatic setting, FOLFIRINOX, mFOLFIRINOX or gemcitabine and albumin-bound paclitaxel represent acceptable treatments with higher response rate than gemcitabine (III, B).
4. After induction chemotherapy (between 4 and 6 months), consolidation with chemoradiotherapy is an option (III, C).

#### Metastatic disease

Taking into consideration the dismal prognosis of these patients, a clinical trial is always a good option if is available.

The chemotherapeutic regimen of FOLFIRINOX has been shown superior to gemcitabine monotherapy in PFS and OS [19]. In the MPACT phase III clinical trial, the combination of gemcitabine and nab-paclitaxel also demonstrated superiority in terms of efficacy for metastatic pancreatic cancer (mPDAC) compared with gemcitabine monotherapy [20]. FOLFIRINOX and gemcitabine/ albumin-bound paclitaxel are both indicated in fit mPDAC patients with ECOG PS 0, 1 and ≤ 75 years old. In fit elderly patients (≥ 75 years) or patients with performance status 2, single-agent gemcitabine or gemcitabine and albumin-bound paclitaxel can be considered based on non-randomized data.

There are conflicting results with oxaliplatin-based chemotherapy in second-line treatment. Napoli trial demonstrated a superior OS, PFS, and RR in patients treated with 5-fluorouracil and nal-IRI compared with patients treated with 5-Fluorouracil alone [22]. This combination is an alternative for a fit patient who progresses to gemcitabine-based chemotherapy when it is available. Unfit patients can be treated with 5-fluorouracil in monotherapy (category 2B). Gemcitabine and albumin-bound paclitaxel is a second-line treatment option for fit patients after a 5-fluorouracil based chemotherapy, with no randomized data.

**Fig. 1:**
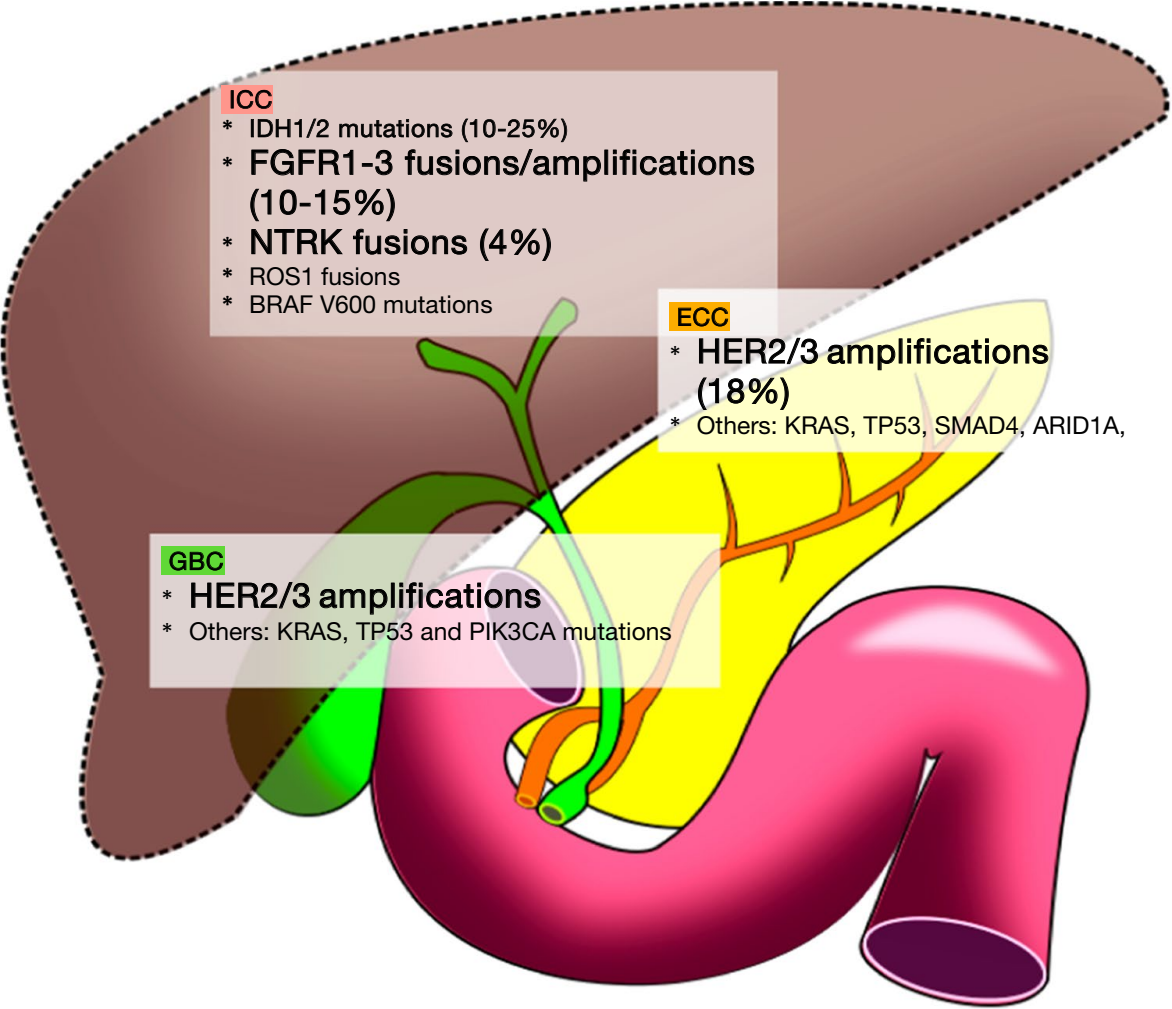
Frequent genomic alterations in biliary tract cancers according to anatomic location. *ICC* intrahepatic cholangiocarcinoma, *ECC* extrahepatic cholangiocarcinoma, *GBC* gallbladder cancer. The most clinically promising drug targets are highlighted in bold type

Pancreatic cancer patients with mutations in *BRCA1* or *BRCA2* with a prevalence between 6 and 7% would be especially sensitive to platinum-based chemotherapy. In this population, we can use FOLFIRINOX, mFOLFIRINOX, gemcitabine with cisplatin or 5-FU and cisplatin. The POLO trial evaluated Olaparib as maintenance therapy for individuals with germline *BRCA1/2* mutations and mPDAC that was stable or responded after at least 4 months of platinum-based chemotherapy. Olaparib arm presented a superior PFS compared with placebo (7.4 m vs. 3.4 m), and some patients treated with Olaparib presented a very durable response lasting in excess of 2 years [23] (Fig. 1), however, in the POLO trial there is no impact in OS.

Patients with mPDAC and microsatellite instability (1–3%) treatment with checkpoint therapy can be considered when is available [24].

**Recommendations**

1. FOLFIRINOX/mFOLFIRINOX and/or Gemcitabine-albumin-bound paclitaxel are standard first-line schedules in metastatic disease in patients with ECOG/PS 0-1 and younger than 75 years old (I, A).
2. In selected patients with ECOG 2 or older than 75 years old, gemcitabine and gemcitabine in combination with albumin-bound paclitaxel can be considered (II, B).
3. The combination of nal-IRI and 5-fluorouracil, is an alternative for a fit patient who progress to gemcitabine-based chemotherapy, when it is available (I, A).
4. Gemcitabine and albumin-bound paclitaxel is a second line treatment option for fit patients after a 5-fluorouracil based chemotherapy (II, B).
5. Current data favor an approach of frontline platinum-based chemotherapy followed by maintenance with Olaparib with mPDAC with germline *BRCA 1* and *BRCA2* mutations (I, A).

### Supportive care

Early and systematic integration of palliative care in oncological care improves clinical outcomes and quality of life (QoL) of patients with advanced pancreatic cancer [25]. In pancreatic cancer, there are five symptoms that must be emphasized: pain, jaundice, gastric outlet obstruction, and nutritional support.

Pain is often the major presenting symptom of the disease. We should indicate opioid medication, considering adjuvant medications in case of neuropathic pain with gabapentin, pregabalin, nortriptyline or duloxetine. In refractory patients celiac plexus neurolysis should be considered, percutaneously, surgically or under endosonographic guidance. It has a success rate of 50–67%, and has demonstrated the superiority of pain relief over analgesic therapy, causing also fewer adverse effects than opioids [26].

For palliation of obstructive jaundice, and its clinical consequences such as cholangitis, pruritus and impaired liver function, we recommend stent placement. Metal stents had a lower risk of recurrent obstruction than plastic stents, without differences in mortality or complications [27]. Plastic stents had a lower duration of patency (above 3 months) than metal stents. The decision to use one versus another should be guided by expected length of survival, disease stage, ECOG, PS and comorbidities. The surgical bypass should only be reserved for those whose stent placement is not possible.

Gastric outlet obstruction, is a frequent symptom as the disease progresses and we recommend the placement of a stent at the duodenum over surgical approach, but the decision should be individualized considering life expectancy, disease stage, ECOG, PS and comorbidities.

All cancer patients should be screened regularly for the risk or the presence of malnutrition. In all patients—with the exception of end-of-life care—energy and substrate requirements should be met by offering in a step-wise manner nutritional interventions from counseling to oral nutrition. However, the benefits and risks of nutritional interventions have to be balanced with special consideration in patients with advanced disease. Pancreatic exocrine insufficiency (PEI) is prevalent among patients with pancreatic adenocarcinoma in up to 64–100% of patients and contributes to fat malabsorption, which leads to maldigestion, steatorrhea, and weight loss. We recommend elastase-1 stool test to diagnose it. Enzyme replacement therapy improved survival in a recent population-based study and starting at doses of 75,000 IU daily are recommended [28].

**Recommendations**

1. Pain management with opioid therapy and celiac plexus neurolysis (I, A).
2. Obstructive jaundice and gastric outlet obstruction should be managed with stent placement (I, A).
3. Patients should be screened with elastase-1 stool test to diagnose PEI and receive enzyme replacement in case of insufficiency (I, A).

### Surveillance

At least 80% of patients will develop local and/or distant disease recurrence after pancreatectomy, often within 2 years. Current evidence for recurrence-focused surveillance after pancreatic cancer resection is limited and contradictory. Although computed tomography positron emission tomography and the serum CA 19.9 can detect preclinical recurrences, there is no evidence that treatment derived from early detection of recurrence increases survival.

**Recommendations**

There is no evidence that regular follow-up after initial therapy with curative intent prolongs survival (IV, D).

## Biliary tract cancer

### Introduction

Biliary tract cancers (BTC), including gallbladder cancer, intrahepatic, perihilar, and distal cholangiocarcinoma, are malignancies that arise from the epithelium of the biliary system. BTC represents a heterogeneous group of cancers with extensive biologic and genetic diversity. With early disease, surgical resection is the preferred option for all types; however, outcomes are poor.

### Epidemiology and risk factors

Represent less than 1% of all cancers. In Spain, during 2019, 2873 new BTCs were diagnosed representing an incidence of 6.1/100.000 [29]. Gallbladder cancer (GBC) is two times more frequent than intrahepatic or extrahepatic cholangiocarcinomas; ampulla of Vater cancer is the less frequent type. Regarding primary liver cancers, intrahepatic cholangiocarcinoma is the second most type after hepatocellular carcinoma.

Etiologic studies on BTCs suggest that risk factors vary by anatomic origin site within the biliary tract. Moreover, incidence rates varied by sex, ethnicity, and age. GBC incidence rates were highest among women and older age groups since gallstones and subsequent cholecystitis are the primary risk incidence factors; approximately a third are attributable to obesity [30], other risk factors are: multiple pregnancies, family history of gallstones, low levels of physical activity, chronic infection by Salmonella (typhi and paratyphi) and Helicobacter (bilis and pylori) isolation. Only 1% of patients with gallstones develop GBC, suggesting that any screening program should be targeted to higher-risk groups. Nowadays, the incidence of GBC is decreasing in the Western world in relation to the increase in routine cholecystectomy [31].

Intrahepatic cholangiocarcinoma (ICC) presents higher incidence rates for males and older ages. It is associated with chronic inflammation of the biliary tree and hepatic parenchyma. Risk factors include chronic liver diseases and cirrhosis, biliary stones, liver infections (hepatitis B and C viruses, liver flukes), bile duct anomalies, some autoimmune diseases, obesity, diabetes, and smoking [32].

Extrahepatic cholangiocarcinoma (ECC) shares some risk factors with ICC. Nevertheless, a prior cholecystectomy increases risk of ECC, but not of ICC. Choledochal cysts or bile duct anomalies have a 1–15% lifetime risk of developing into GBC and cholangiocarcinoma. The risk of finding malignancy is higher when the diagnosis is made in adults or older age. Surgical resection, at the moment of diagnosis, is indicated to prevent malignancy.

### Clinical diagnosis and staging

BTCs common classification is based on the anatomical site of origin: ECC are those CCAs arising from the common bile duct and its tributary branches; ICC (around ~ 30%) are those originating from the small ducts within the liver [33]. ECC can develop from the second-order of the bile ducts to the junction of the cystic duct and the common hepatic duct (perihilar, pECC, ~ 50%) or can arise from the common bile duct between the cystic duct origin and the ampulla of Vater (distal, dECC, ~ 20%).

Diagnosis of BTC is challenging, since, in most cases, patients can remain asymptomatic for a long period during early stage-disease or symptoms can be inspecific [34]. In ECC symptoms such as biliary obstruction and cholangitis are more frequent and precocious than in ICC and GBC. In consequence, more than 60% of BTC are diagnosed at an incurable advanced stage, and in less than 40% of all cases, a surgical resection is an option.

Early-stage disease is usually diagnosed through pathology workup after routine surgery (cholecystectomy) or as an incidental finding during a routine radiology test.

**Recommendations**

1. The initial workup for those patients with suspicion of a BTC should include a blood test with liver function parameters (CEA and Ca 19.9 blood levels could be considered but not for diagnostic purposes) and a MDCT, with or without contrast, of the chest, abdomen, and pelvis (II, A) [35, 36].
2. A magnetic resonance cholangio-pancreatography (MR-CP) can assess the presence of satellite and distant lesions in the liver and invasion of biliary tract, major vessels, and nearby lymph nodes (II, A).
3. The role of PET scan has not been well established although may be useful for the detection of regional lymph node and distant metastases (III, C).
4. Assessment of hepatic reserve is mandatory in GBC and ICC when surgical resection is indicated. There are no pathognomonic imaging or pathologic features definitely associated with ICC. Nonetheless, LI-RADS can be useful in distinguishing between ICC and hepatocellular carcinoma (HCC) (II, A).
5. For ICC patients undergoing resection biopsy is usually not necessary and an esophageal-gastroduodenoscopy and a colonoscopy could be recommended in some cases (IIIB, C).
6. In ECC endoscopic retrograde cholangiopancreatography (ERCP) can be performed for complete imaging of the duct, brush cytology and stenting of obstruction when required (II, A).

### Molecular pathology

#### Prevalent genomic alterations

Molecular cancer subtyping ultimate goal is to uncover potential targets amenable to become drug targets. Potentially targetable genetic alterations occur in around 40–60% of BTC patients. Interestingly, these aberrations distribution is according to the anatomic location: in ICC are found mostly mutations in IDH1/2, BAP1, and ARID1A and FGFR rearrangements, while in ECC are more frequently found novel fusions in PRKACA/PRKACB and mutations in ARID1B (see Fig. 1) [37].

Alterations in DNA repair genes (MSH6, BAP1, ATM, MLH1, MSH2, BRCA1 and BRCA2) are relatively frequent: up to 16% of ICC and 45% of ECC display mutations in these genes [38].

In GBC the most frequently mutated genes include KRAS, TP53, ERBB3, and PIK3CA, whereas no mutations have been identified in IDH1 or IDH2.

The most frequent actionable alterations are IDH1/2 gain of function mutations and FGFR2 fusions/rearrangements, found in around 5–36% and 8–25%, respectively, of CC cases, mostly ICC. Amplification of HER2 (3–19%) is more frequent in GBC and ECC than in ICC.

BRAF (~ 5%) and BRCA2 mutations (~ 5%), fusion genes involving the genes NTRK1/2/3 (4%), ROS (8–9%), ALK (3%) and mismatch repair deficiency (dMMR) and/or high microsatellite instability (MSI-H) (3% in ICC, 2% ECC and 6% in GBC) are quite rare in BTC [39].

The main targetable genetic aberrations (IDH1 and BRAF mutations, FGFR fusions, HER2 amplification) can be identified in circulating tumour DNA (ctDNA) in around 20% of BTC patients.

#### Molecular classification

Several molecular classifications of CC have been proposed (no molecular classification of GBC has been published thus far); nonetheless, none has yet been translated into the clinical setting [35].

**Recommendations**

For patients with MMR/MSI-H tumors and BRCA 1/2 mutations consider referral to genetic counseling and germline testing (III, B).

### Treatment

#### Localized disease

Surgical resection with free margins is the only curative treatment for BTC and is individualized based on tumour location. Only 20% of patients are candidates for curative surgery. The most relevant prognostic factors are margin status and lymph node metastases.

##### Adjuvant therapy

Despite optimal surgical resection around 60–70% BTC relapse. Since the last update of guideline three randomized phase III clinical trials have compared different chemotherapy regimens with observation following surgery.

The BILCAP trial randomised 447 patients with ECOG performance status 0–2 to capecitabine 1250 mg/m^2^ twice daily on days 1–14 of a 3 week cycle, for 24 weeks or observation [40]. Capecitabine initiation was required within 16 weeks of surgery. All types of BTC were included and the proportion of patients with N1 disease and R1 resection was 47% and 38% respectively. After a median follow up of 60 months the primary endpoint was not met, mOS in the ITT population was 51.1 months in the capecitabine arm compared to 36.4 months in observation arm (HR 0.81, 95% CI 0.63–1.04; *p* = 0.097). In a protocol-specified sensitivity analysis, adjusting for nodal status, grade, and gender, a significant difference in OS was found (HR 0.71, 95% CI 0.55–0.92; *p* = 0·010). This significant difference in OS was also described in the per-protocol analysis (HR 0.75, 95% CI 0.58–0.97; *p* = 0.028). The RFS was significant longer for capecitabine (24.4 months vs. 17.5 months, HR 0.75, 95% CI 0.58–0.98; *p* = 0.033). The capecitabine dose is still a matter of debate and there is no consensus on the optimal dose.

There is a lack of RCT that assesses the benefit of adjuvant radiotherapy. Several small trials [41] and meta-analysis [42] have suggested a survival benefit for chemoradiotherapy in eCCA and GBC with R1 resection or node-positive disease. The optimal fractionation schedule, dose and concurrent chemotherapy regimen is not established.

Neoadjuvant therapy is considered an experimental approach and should be used only in the context of a clinical trial.

**Recommendations**

1. After curative resection of BTC, all patients should receive adjuvant therapy with capecitabine for 6 months (I, B).
2. Chemoradiotherapy may be considered in eCCC or GBC with R1 resection or BTC with node-positive disease (II, B).

#### Locally advanced disease

There is a lack of specifically dedicated RCT in unresectable locally advanced BTC. Systemic chemotherapy is the initial therapy of choice in this setting. The available evidence to support this recommendation comes from large randomized trials in the advanced disease that included a subgroup of patients with unresectable locally advanced tumours. The same therapeutic principles as described below in metastatic disease must be followed. The combination of cisplatin and gemcitabine is recommended whenever possible [43]. Other alternative regimens are based on fluoropyrimidines or oxaliplatin. After an initial period of at least, 3–4 months of systemic chemotherapy locoregional therapy can be discussed in a multidisciplinary tumour board. Different radiotherapy techniques may be considered, including chemoradiation or stereotactic body radiation therapy (SBRT), depending on local availability. Also, radiofrequency ablation, transarterial chemoembolization or radioembolisation with 90Y-microspheres may be an alternative in ICC. Reassessment for resectability should be perform along therapy evolution. If tumor downstaging amenable to resection is achieved salvage surgery should be considered. Orthotopic liver transplantation should not be offered outside a clinical trial in high-volume and experienced transplant centers.

**Recommendations**

1. Upfront systemic chemotherapy is the treatment of choice in unresectable locally advanced BTC (I, B).
2. Locoregional therapy can be considered only after an initial period of at least 3–4 months of systemic chemotherapy (II, B).

#### Metastatic disease

Randomized controlled trials conducted in patients with advanced BTC demonstrated that systemic chemotherapy prolongs survival over best supportive care [44, 45]. A pooled analysis of 104 trials including 2810 patients observed that gemcitabine and platinum-based regimens were associated with increased response and tumor control rates [46]. The improved efficacy of this chemotherapy regimen was further supported by the results of 2 randomized studies that established gemcitabine-cisplatin as the treatment of choice in this setting [43, 47]. The ABC-02 study randomized 410 patients with advanced or metastatic cholangiocarcinoma, gallbladder, or ampullary cancer to receive gemcitabine with or without cisplatin. Overall survival (OS) was significantly longer in the cisplatin-gemcitabine group as compared to the gemcitabine group (median of 11.7 vs. 8.1 months; HR 0.64; *p* < 0.001) [43]. Progression-free survival (PFS) (8 vs. 5 months, HR 0.63, *p* < 0.001) and tumor control rate (81.4% vs. 71.8%, *p* = 0.049) also favored the cisplatin-gemcitabine group. A Japanese randomized phase II study reported similar results [47]. A meta-analysis of both trials indicated that benefit from the combination was independent of age, gender, tumor stage (locally advanced vs. metastatic), prior therapy (surgery vs. stent) and primary tumor site (intra- vs. extra-hepatic vs. gallbladder vs. ampullary), although subgroups least likely to benefit were patients with ampullary tumors and poor performance status [48].

In the second-line setting, the ABC-06 randomized study also demonstrated a meaningful survival improvement with chemotherapy [49]. This trial randomized (1:1) 162 patients with advanced biliary tract cancer (BTC) and disease progression after prior treatment with cisplatin-gemcitabine to receive mFOLFOX or active symptom control (ASC) only. Median OS was 6.2 vs. 5.3 months for the mFOLFOX and ASC arms, respectively, and the 12 month OS-rate was 25.9% vs. 11.4% (HR 0.69 adjusted for platinum sensitivity, albumin, and stage; *p* = 0.031).

More importantly, an improved understanding of the molecular bases of BTCs has enabled the identification of new targets for therapy with some recent encouraging results. In this context, a phase 3, randomized, double-blind study (ClarIDHy) randomized (2:1) 185 patients with IDH1-mutant cholangiocarcinoma, previously treated with up to 2 treatment regimens, to receive a mutated IDH1 inhibitor, ivosidenib (AG-120), or placebo [50]. PFS was significantly improved with ivosidenib compared with placebo (median of 2.7 vs. 1.4 months, HR 0.37; *p* < 0.0001). PFS at 6 and 12 months was 32% and 22% for ivosidenib-treated patients, whereas there were no patients in the placebo group free from progression for 6 months or more. A trend towards an improved OS for the ivosidenib group was also observed (median of 10.8 vs. 9.7 months, HR 0.69, *p* = 0.060), although the cross-over design (57% of patients in the placebo group received ivosidenib upon progression) limited the ability of this trial to properly address this endpoint. Pemigatinib, a small molecule inhibitor of fibroblast growth factor receptor (FGFR) 1, FGFR2 and FGFR3, received accelerated approval in April 2020 in the USA for the treatment of previously treated advanced or metastatic cholangiocarcinoma with FGFR2 fusions [51]. This decision was based on results from a single-arm, phase 2 study (FIGHT-202) that enrolled 146 patients, 107 with FGFR2 fusions or rearrangements, 20 with other FGF/FGFR alterations, and 18 with no FGF/FGFR alterations. An objective response was observed in 38 patients with FGFR2 fusions or rearrangements (33%), including 3 complete responses. Most frequent grade 3–4 AEs were hypophosphataemia (12%), arthralgia (6%), stomatitis (5%), hyponatraemia (5%), abdominal pain (5%), and fatigue (5%). Several FGFR2 inhibitors (infigratinib, TAS120, pemigatinib) are currently being evaluated in several ongoing randomized trials in advanced cholangiocarcinoma with FGFR rearrangements, versus cisplatin-gemcitabine in the first-line setting. Results are awaited with great interest as they may be practice-changing in the near future.

BRAF mutations are encountered in 1–22% of intrahepatic cholangiocarcinomas. The ROAR basket trial (NCT02034110), included different tumors with BRAF V600E mutation that were treated with dabrafenib and trametinib [52]. Preliminary results in the BTC cohort (35 patients, 80% pretreated with > 2 prior lines of chemotherapy) were encouraging, with a response rate of 42% and a median survival of 11.7 months. Other ongoing trials (NCT01713972, NCT01902173) are also assessing dual MEK/BRAF inhibition in patients with BRAF‐mutant cholangiocarcinoma and shall help elucidate the role of this strategy in these patients. Other potential targets currently being explored include Her2 amplification that is not uncommon in gallbladder cancer, but only case reports are available to date. Additional targeted approaches to be considered in certain subgroups of patients with advanced BTC include some recently approved drugs with molecularly-driven, tumor-agnostic indications. Such is the case for pembrolizumab for tumors with microsatellite instability (< 1% of cholangiocarcinomas) or high tumor mutational burden (TMB > 10 mutations/Mb) [53, 54], or TRK-inhibitors (larotrectinib or entrectinib) for tumors with NTRK rearrangements (< 5% of cholangiocarcinomas) [55, 56]. Only small numbers of BTC patients have been, however, included in these tumor-agnostic trials. Overall, these results highlight the growing role and clinical relevance of tumor mutational profiling in the management of advanced BTC. However, none of these targeted therapies have been approved to date by the European Medicines Agency (EMA).

**Recommendations**

1. Gemcitabine and cisplatin is the treatment of choice as first-line therapy in fit patients with advanced BTC (I, A).
2. Oxaliplatin may be an alternative to cisplatin in patients with impaired renal function, and monotherapy with gemcitabine or fluoropyrimidines may be considered in frail patients (II, B).
3. mFOLFOX is the preferred treatment regimen in the second-line setting for fit patients with advanced BTC and no severe residual neurotoxicity from prior therapy (I, A). Fluoropyrimidines may be used as monotherapy in this setting in patients with significant cumulative neurotoxicity (III, C).
4. A comprehensive tumor molecular characterization shall be pursued where available in patients with advanced BTCs, as certain genetic alterations, such as IDH1 mutations (I,A), FGFR2 fusions (II, B), BRAF mutations (II, B), microsatellite instability (II, B), high-TMB (II, B), and NTRK rearrangements (II, B), among others, may benefit from specific targeted therapies. Enrolment of these patients in clinical trials is highly encouraged.

### Supportive care

Early implementation of supportive care and active symptom control is very relevant for patients with advanced, recurrent or metastatic disease as it has a major impact on patient’s quality of life and may also extend survival [50]. The focus should be placed in symptom management (pain, nausea, constipation, diarrhea, anorexia, weight loss, anxiety, depression) and most common disease-related complications, such as infections (i.e. cholangitis) and biliary or duodenal obstruction. Biliary drainage and endoscopic procedures are commonly required and may be planned in some patients with recurrent complications. A metal stent is preferred in patients with a life expectancy of > 3 months. Percutaneous transhepatic biliary drainage is recommended if endoscopic stenting is not feasible.

#### Follow-up

There are currently no data to support a specific surveillance schedule for BTCs treated with curative intent, nor is there any evidence to demonstrate that regular follow-up influences outcome. Nevertheless, most physicians do regularly follow these patients, most commonly with 3-monthly visits during the first 2 years after therapy, and 6-monthly thereafter up to 5 years, including clinical examination, laboratory tests (including LFTs and lactate dehydrogenase), tumors markers (CEA, CA19-9) and a body CT scan every 3–6 months (IV, D).
